# Robotic assisted fixation of sacral fractures: A pilot study

**DOI:** 10.1097/OI9.0000000000000046

**Published:** 2019-12-06

**Authors:** Yoram A. Weil, Amal Khoury, Rami Mosheiff, Leon Kaplan, Meir Liebergall, Josh E. Schroeder

**Affiliations:** Department of Orthopaedics, Hadassah Hebrew University Medical Center, Jerusalem, Israel

**Keywords:** iliosacral screw, robotic surgery, sacral fracture

## Abstract

**Objectives:**

Sacral fractures that require fixation are a challenge for the orthopaedic surgeon. Due to anatomical consideration, implant insertion is not risk free, and requires a steep learning curve. A robotic system has been successfully used in pedicle screws insertion and can be also used for iliosacral screws. The aim of the study was to demonstrate the use of the robot in the treatment of unstable sacral fractures.

**Design:**

Retrospective case series.

**Setting:**

An academic level I trauma center.

**Patients:**

Fourteen patients with sacral fractures were eligible for robotic assisted treatment. These included 9 high-energy fractures, 4 osteoporotic fractures, and 1 pathological fracture.

**Intervention:**

Fixation constructs included iliosacral screws, transiliac screws, lumbopelvic fixation, sacroplasty, or a combination of the above techniques. A Renaissance robot was mounted on a multidirectional bridge that was attached to the patients spine and implant trajectories were planned either on preoperative or intraoperative 3D scans. Guide wires were inserted percutaneously and screws were placed subsequently.

**Main outcome measurements:**

Accuracy of implant placement, operating room and fluoroscopy time.

**Results:**

Mean patient age was 36 (17–84), and number of screws, including iliosacral and pedicular ranged 1–14 per patient (average 4.25). Mean operative time was 150 minutes (range 90–300). Average fluoroscopic time was 18 seconds (7–42) for 2D and 40 seconds (12–72) for 3D imaging. All fractures healed, no hardware failure was observed. All hardware was always within bony confines, and no procedure-related neurological deficits were observed.

**Conclusion:**

Robotic assisted fixation of sacral fracture is a safe and reproduceable method, allowing precise and accurate implant placement.

## 1 Introduction

Unstable posterior pelvic ring injury that requires operative fixation is a major challenge for the trauma surgeon. Vertical instability and comminution are often encountered and may compromise fixation success.^[1]^

Most commonly, conventional, biplanar fluoroscopy is used for screw insertion, and during the past decades percutaneous iliosacral screws have become a common tool for fixation of posterior ring injuries, including vertical sacral fractures.^[2]^

As unstable sacral fractures are concerned, several fixation methods, after closed or open reduction, have been proposed. These include iliosacral screws, posterior tension band plating,^[3]^ transiliac, trans-sacral screws^[4]^ and lumbopelvic fixation,^[5]^ as well as combinations of all the above. However, these techniques are not risk free, mainly due to anatomical considerations and fracture patterns. The anatomical safe corridor that allows for the fixation of the ilium to the first sacral vertebral body is a major concern that needs to be assessed and is limited in some patients due to sacral dysmorphism.^[2,6]^ Also, for an S2 iliosacral screw, this corridor tends to be even narrower^[6]^ and anatomically varied among individuals.^[7]^

Besides potential nerve injury, the tenuous soft tissue envelope around the sacrum makes a formal open reduction, especially with additional lumbo-pelvic instrumentation a high-risk procedure for soft tissue complications, skin breakdown, and infections.^[8]^

Despite the rising popularity of percutaneous posterior pelvic ring fixation, there is still a high amount of described hardware misplacement ranging from 10% to 15% and even higher,^[9,10]^ leading to mechanical and neurological sequalae. This high misplacement rate created the need for a more accurate technique of screw placement.

The use of computer-assisted surgery, namely computerized navigation, has been described for the insertion of iliosacral and other pelvic screws with a variable degree of success.^[11,12]^ However, even after almost 2 decades since its introduction, it has not become a common practice in the orthopaedic trauma community. Possible explanations of this unacceptance are related to technical issues associated with the requirement for direct line of sight when using optical systems, cumbersome setup, unfriendly user interface, and a steep learning curve.^[13]^

In spine surgery, placement of screws in pedicles with a narrow safe corridor is a part of the everyday practice. The malposition rate can vary between 3% and 15%^[14,15]^ according to level of the spine and deformity level. Therefore, image-guided systems have been more commonly used in spine surgery than in general and pelvic orthopaedic trauma. One of these devices is a hexapod robot that has been introduced to improve the accuracy of pedicle screw placement.^[16]^ This robotic device uses either preoperative or intraoperative imaging data and can direct a mechanical arm to the trajectory of a screw based on the intraoperative planning of a 3D image. Accuracy results with the use of the robot in spine surgery are considered to be excellent with over a decade of experience.^[17]^ Technically, it is possible to insert iliosacral screws with the aid of the robot. Furthermore, accurate insertion of hardware for both spine and pelvis fixation can result in less invasive surgery, thus reducing the morbidity associated more extensile approaches, such used for lumbopelvic fixation.^[8]^ The aim of this study is to demonstrate the use of a robotic system in the fixation of unstable sacral fractures using either iliosacral screws alone or in conjunction with lumbopelvic fixation in a percutaneous mode.

## 2 Patients and methods

Fourteen patients with sacral fractures were treated with robotic assisted iliosacral and/or lumbopelvic fixation between 2014 and 2018. These included 9 high-energy traumatic fractures, and 5 low-energy osteoporotic fractures out of which 1 was a pathological fracture. Fixation included iliosacral screws, trans-iliac-trans-sacral screws, lumbopelvic fixation, and 1 case of cement injection only. The mechanisms, age, and fixation types are specified in Table 1. *Indication for surgery*: the 4 nontraumatic fractures were treated surgically after a failure of nonoperative treatment due to intractable pain and decreased ambulatory capacity. For traumatic fractures, indication for surgery included unstable fracture pattern (vertical shear), and radiographic signs of instability such as L5 transverse process avulsion and/or zone 2 sacral fractures.^[1]^ Fixation constructs were determined according to the degree of instability. Severely displaced pelvic fractures, or pelvic fractures associated with vertebral fractures, were treated with lumbopelvic fixation.

**Table 1:**
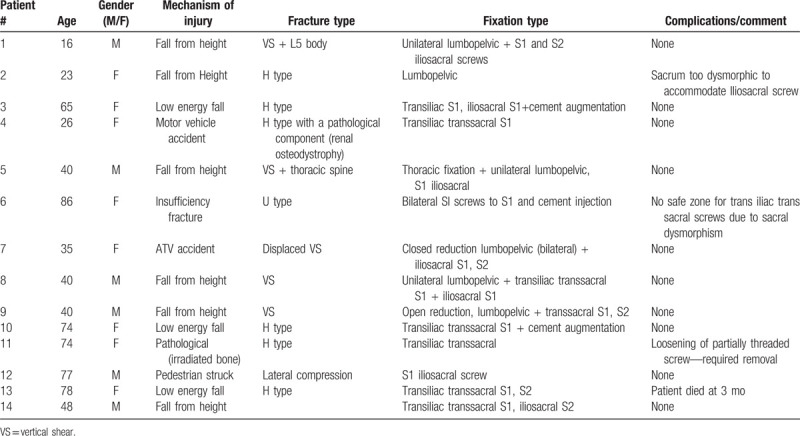
Patient demographics, mechanism of energy, and surgery type.

### 2.1 Surgical technique

Surgical technique relied on the image platform used for preoperative planning.

### 2.2 The Renaissance robotic system

The Renaissance (Mazor Robotics Inc, Orlando, FL) is a commercially available system used for insertion of pedicle screws. Its core component is a hexapod robot that can deform in 6 degrees of freedom, connected to a drill sleeve.

The robot is mounted on a plastic (PEEK) bridge affixed to the operating room table, and to a spinous process adjacent to the level of fixation. The surgical act of robotic screw insertion involves directing the robotic arm into the desired screw trajectory planned on a computer station using preoperative or intraoperative computed tomography (CT) images. The process commences with planning the chosen screw trajectory on the preoperative CT images (axial, coronal, and sagittal cuts) using a dedicated computer station. The next step involves a registration process where intraoperative 2D fluoroscopy is used to verify the patient anatomy to the preoperative CT scan using a propriety 2D to fluoroscopic merge. Alternatively, an intraoperative 3D fluoroscopy using different commercially available 3D scanners (such as O-arm, Zeego, and others) can be used to create the images for screw planning, obviating the need for the registration process. Once the screws’ trajectories are planned, the operator at the computer station attached to the robot indicates the position on the plastic bridge (the table mounted frame) to mount the robot on, and “sends” the robot to travel to the desired entry point. After the robotic motion is stopped—the surgeon uses specialized surgical instruments that pass through the robotic arm to incise the skin, pass trocars, and drill sleeve and “blindly” drill the screw trajectory according to the depth and length dictated by the plan. The reader can access the manufacturer's website for further details.^[18]^

As described above, the surgical robot requires a three-dimensional dataset that can be obtained either preoperatively using a CT scan or intraoperatively using a 3D scanner producing CT-like images. For the first option—a propriety CT to intraoperative 2D fluoroscopic merge algorithm using 2 fluoroscopic images only was done for image registration. For the second option—this step was not required since real-time CT like 3D images were obtained. These options are further specified below.

In 8 cases, where fractures were minimally displaced and therefore a change in the surgical anatomy was not anticipated, a CT scan was performed preoperatively and uploaded to the Renaissance (Mazor robotics, Israel) robot workstation. At the workstation after processing of the images—the user can browse through axial, sagittal, and coronal reformats of the CT scan. A virtual screw trajectory is drawn on all planes and can be fine tuned in rotation, entry point, and length. The station warns if the planned screw is not reachable by the mobile robot. The desired screw trajectories are stored in the computer's memory. Patients were placed prone on a radiolucent table. A PEEK Multiple Directional Bridge (MDB) was mounted on the surgical table connected to a pin fixated on a lumbar vertebra spinous process or on the posterior superior iliac spine. Intraoperative registration of 2 fluoroscopic images: an AP image and a 60 degrees’ lateral were performed—for verification purposes—it should be noted that these images were not used for screw planning per se. The robot was mounted on the MDB and was sent to the required trajectories. A set of specialized instruments including PEEK connectors for off-center trajectories (such as iliosacral screws) were mounted on the bridge if needed (as dictated by the station), and specialized drill sleeves were inserted through the robotic arm after it reached its destination for each screw. A percutaneous incision was performed and either a drill (pedicle screw) or a 2.8 mm guidewire (iliosacral screw) was introduced after drilling with a 3.0 mm drill bit. We preferred to use a 3.0 mm drill bit first since the propriety cannula of the Mazor system, has this internal diameter and the drill bit is more rigid than a long 2.8 mm guidewire, thus reducing the risk of wire deflection or bending during insertion. For the iliosacral screw placement—it should be stressed that guide pins were drilled through the robotic arm manually and haptic feedback of the sacral ala and sacral vertebral body was similar to other percutaneous, conventional techniques.^[2]^ Also, the 3.0 mm drill bits were calibrated with the designated drill sleeves, and therefore the depth of each planned wire was reached, without the use for fluoroscopy for each individual wire. After all guide wires were placed, verification radiographs were taken. Cannulated screws were placed over the guidewires/drill-bits using the standard surgical technique. Fixation was completed, and postoperative care was given depending on the fracture type. All patients underwent postoperative CT for verification of implant placement.

In 6 patients with displaced fractures, intraoperative 3D scan was performed using the Artis-Zeego (Siemens, Germany) motorized stationary imaging system in a hybrid room, after reduction of displaced fractures. Acceptable reduction was defined as less than 5 mm of displacement and restoration of correct height as seen on axial and coronal cuts provided by the scan (Fig. 2, top panels).

**Figure 1:**
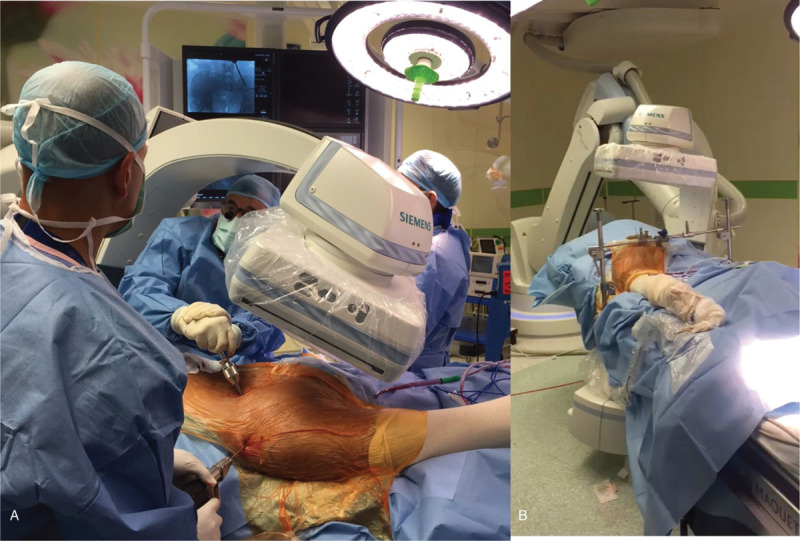
A 34-yr-old woman with an unstable, displaced fracture is being operated on. (A) A closed reduction maneuver is attempted with a 5 mm shanz screw inserted into the PSIS and the hemipelvis is pushed anteriorly by applying axial load combined with longitudinal traction. A provisional 2.8 threaded wire is inserted to maintain the reduction. (B) The 3D fluoroscope is capturing a 3D scan while the surgical team is away.

**Figure 2:**
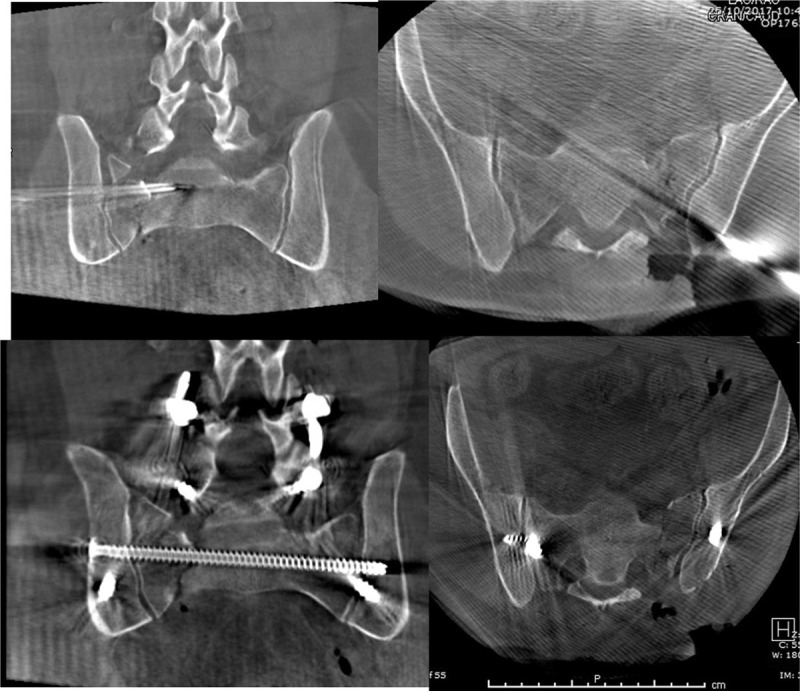
Intraoperative images during surgery—top 2 figures demonstrating intraoperative 3D scans after open reduction and provisional k-wire fixation, while bottom 2 images represent intraoperative 3D scan after definite fixation with lumbopelvic fixation and a trans-iliac trans sacral S1 screw. All implants (spinal and pelvic) were inserted with robotic guidance.

**Figure 3:**
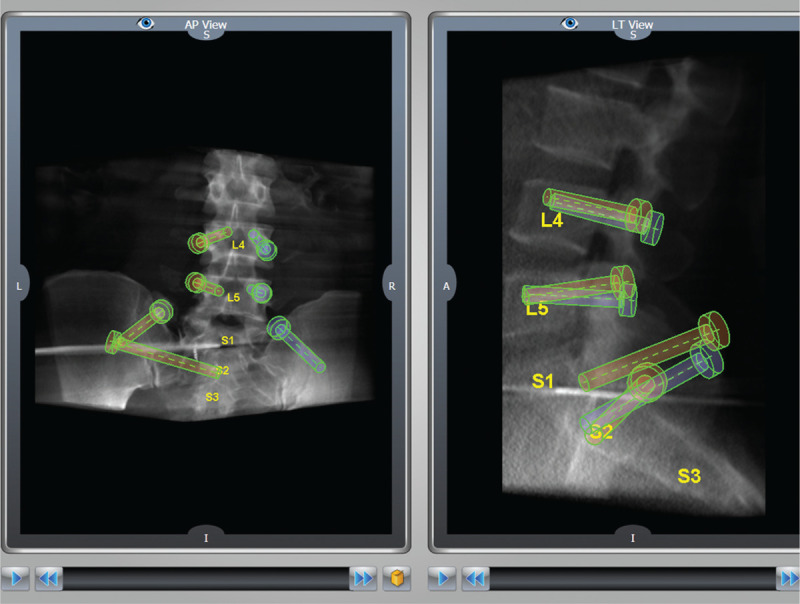
A 3D reconstruction of the planned lumbopelvic fixation of the above fracture. This planning will guide the robotic arm later on.

**Figure 4:**
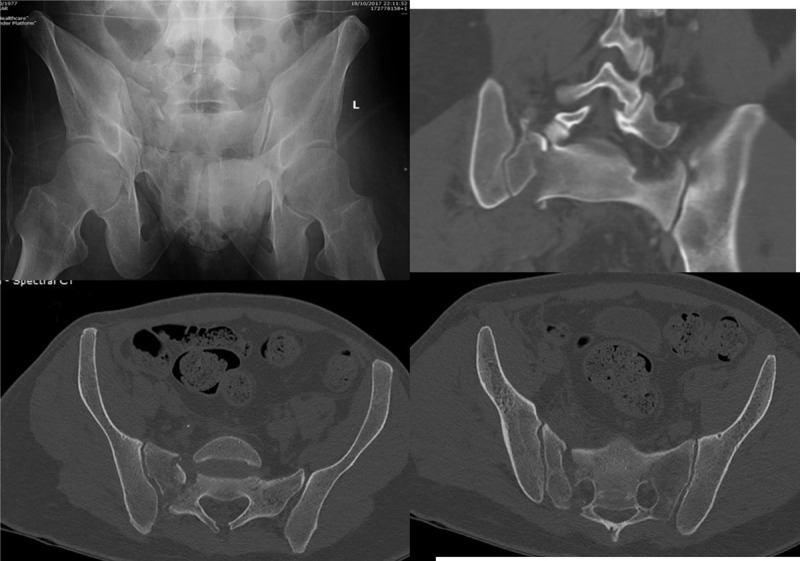
A displaced vertical shear fracture of a 40-yr-old laborer who fell from height—the outlet (top left), view, coronal, and axial CT demonstrate severe displacement. The reduced fracture is seen in Figure 2.

This scan was transferred to the Mazor station. Implant trajectories were planned as done with the previous method (Fig. 3). Displaced fractures were reduced with a closed manipulation in the operating room, and held in place with a provisional kirschner wire if needed (Figs. 1A and 2), prior to the intraoperative scan. One severely displaced sacral fracture (3 cm of vertical displacement) required an open reduction using a standard posterior approach (Figs. 2 and 4).

After planning, implants were placed as described above. In the cases operated in the hybrid room—intraoperative 3D scan was performed after placement of implants to verify correct implant position. The other patients underwent postoperative CT scans.

In 10 out of 14 patients, intraoperative sensory and motor-evoked nerve potentials nerve monitoring were used throughout the case.

## 3 Results

**Figure 5:**
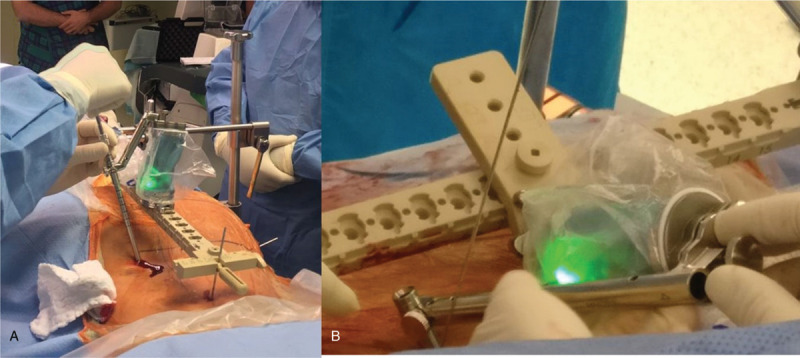
(A) The robot (green) had completed its travel over the plastic bridge into a desired pedicle screw trajectory. The skin is incised and the guidewire is drilled through the sleeve connected to the robot. (B) With another adapter the robot is directed more cadually and laterally in order to start the trajectory of the iliosacral screw/s.

**Figure 6:**
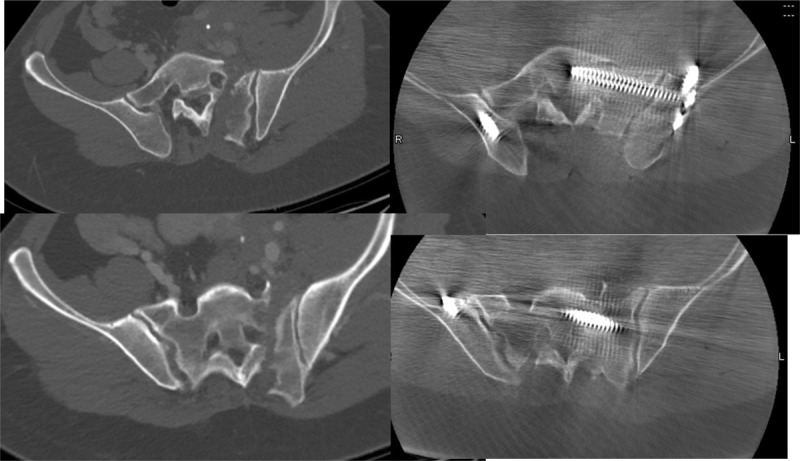
The preoperative CT scans (2 left panels) compared with the intraoperative 3D fluoroscopic scans (2 right panels) demonstrating improved albeit imperfect reduction with adequate implant position.

**Figure 7:**
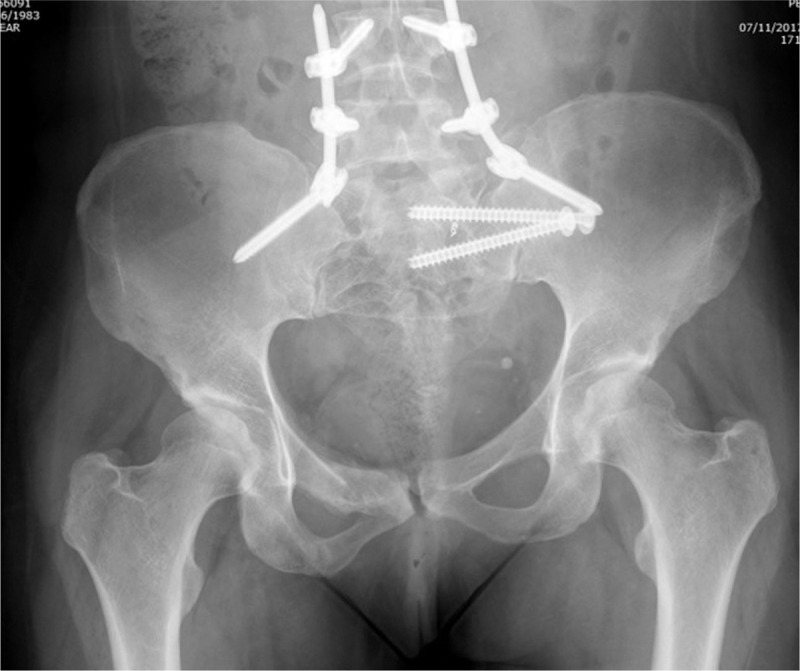
The complete fixation as seen in a postoperative radiograph obtained at 3 mo postoperatively. The patient is pain free and weight bearing.

Mean patient age was 36 (range 17–84). Fractures type included traumatic fractures due to motor vehicle accidents and falls including vertical shear and H-type fractures (9 patients), 3 low-energy insufficiency fractures and 1 pathological fracture in an irradiated bone. Number of screws were between 1 (iliosacral) to 14 (lumbopelvic) (average 4.25). Operative time including positioning, setup of robotics, and neuromonitoring ranged from 40 to 300 min (average 150 min). Average fluoroscopic time in the cases treated with conventional fluoroscopy was 18 seconds (range 7–42 s). With the use of the 3D fluoroscopy that included also between one to three 3D scans the average fluoroscopic time was 40 seconds (range 12–72 s). All patients underwent either a postoperative CT scan or intraoperative 3D scan after implant placement. In all these cases, postfixation imaging demonstrated confinement of the implants within the bone without breaching of the neural foramina, sacral canal, pedicles, or anterior sacral cortex. A case of a displaced high-energy sacral fracture is demonstrated in Figures 1, 3, 5–7. While a second case with severely displaced sacral fracture that required an open reduction is depicted in Figures 2 and 4.

No wound or soft tissue complication was seen, and patients were discharged 1 to 9 days after admission to home or rehabilitation units, depending on associated injuries. For the patients with lumbopelvic fixation (5 patients) no soft tissue problem or skin breakdown was observed until the latest follow-up (range 9–18 mo).

At latest follow-up (range 6–28 mo, mean 15 mo) all fractures have healed and there were no secondary displacements of the fractures as assessed by plain AP, inlet, and outlet pelvic radiographs obtained in each visit. In 1 patient with an osteoporotic fracture a partially threaded screw backed out and had to be subsequently removed after fracture healing, without further sequelae.

In 1 patient an S1 nerve root weakness was diagnosed preoperatively but did not improve after surgery. No change in intraoperative somatosensory or motor function was noted during neruo-monitoring in any of the patients including the latter one. Of note that in none of the patients either intraoperative or postoperative neurologic complications were observed.

## 4 Discussion

The role for minimally invasive fixation of posterior pelvic ring injuries is now well established and percutaneous fixation with iliosacral screws had become the standard of care since their introduction.^[2,10]^ Two main issues with conventional fluoroscopy remain unsolved—the high amount of radiation required for percutaneous insertion and the high screw misplacement rate. The latter can reach to as high as 30% averaging between 10% and 15% in published case series.^[2,10,19]^ Despite that, some authors claim to reduce misplacement rate using a lateral view starting point technique.^[20]^ As for the fluoroscopic time, although rarely reported in published series, cadaveric data demonstrate a time frame of minutes of continued fluoroscopic radiation time.^[21]^ It is now well established that orthopaedic surgeons are at an increased health risk following radiation use,^[22]^ and therefore any decrease in intraoperative radiation can be of a significant benefit. The use of the robotic system in general tends to decrease intraoperative imaging in certain cases. In cases where previously obtained CT scans were used for screw insertion, the radiation during surgery was limited to a few fluoroscopic shots for the registration process. A different situation regarding intraoperative radiation exposure of the patients occurred when intraoperative CT scans were used. Even with that, the surgical team in our series was well protected from the radiation source in a built-in radiation shielded part of the hybrid operating room. However, radiation dosimetry for the patients and the team were not measured in this study and these should be further tested in the future.

The accuracy of image-guided computer-assisted surgery has been a matter of discussion in the literature. As the first reports of computer-assisted navigation using standard, multiplanar two-dimensional fluoroscopy were published, there was enthusiasm with the accuracy of this technique.^[11,21]^ However, later reports demonstrated that with the use of a standard 2D fluoroscope, no real advantage in terms of screw misplacement is achieved over conventional fluoroscopy.^[19,23]^ These findings are consistent with the enormous variability of the sacral anatomy, which is often hard to assess with two-dimensional imaging.^[24]^ The emerging use of 3D intraoperative imaging had allowed us to assess complex anatomies such as sacral dysmorphisms. Additionally, the availability of intraoperative 3D imaging allowed us to operate on displaced fractures and to obtain real-time data during and after fracture reduction. This data, in turn, was used for robotic insertion of implants. Indeed, the use of intraoperative 3D imaging has been shown to improve iliosacral screw placement.^[19,25]^

In severely unstable sacral fractures, the use of lumbopelvic fixation has been advocated, due to its mechanical superiority over iliosacral screws alone which are more prone to fail in vertical shear severely displaced sacral fractures.^[1,5]^ Despite its mechanical advantage, lumbopelvic fixation has not become a very common method of fixation mainly due to its unacceptably high soft tissue complication rate,^[8]^ and alternative techniques such as transiliac transsacral screw fixation became more popular.^[4]^ However, when converting to percutaneous techniques, the complication rates reduced dramatically.^[26]^ The addition of both intraoperative 3D imaging and a guiding robot makes this procedure a relatively simple one, reducing both surgical exposure and radiation time. In our cases, although only 4, neither mechanical complication or any wound problem occurred, hinting that minimally invasiveness coupled with advanced imaging has the potential for improvement of outcome associated with lumbopelvic fixation. It is of note that the authors felt that the lumbopelvic construct had sufficient stability obviating the need for supplemental anterior fixation. Supporting this notion, is the fact that none of the fractures redisplaced during follow-up.

A limitation that can deter surgeons for adapting the robotic technique is the steep learning curve associated with its application. In our center, one of the senior authors was extensively familiar with the robotic setup and technique and together with technical support throughout the surgery with the robotic company representative setup time was limited to 30 to 50 minutes for each case. However, operating time was still relatively long. Newer generations of robotics are emerging with simpler and easier installation, abolishing the need for a reference bridge and facilitating these procedures.

The limitation of this study is its small and retrospective nature, and the mixture of pathologies ranging from low-energy insufficiency fractures to high-energy unstable sacral fractures, was well as the lack of a control group. For the first issue, the authors thought that as at first, incorporating new technology will be best tried in stable situations. However, even for nondisplaced fracture, in elderly female patients the safe corridor for transiliac fixation can be narrow and challenging. We also could not compare this group of patients to other patients operated in our center using a more conventional technique due to different indications (vertically vs rotationally unstable fractures) and lack of sufficient data on the latter group. However, as robotic technology will develop, further studies with higher power will enable us to further evaluate their potential benefit.

Another limitation of this technique is the use of a prone position—where some patients who require anterior fixation must be repositioned to the supine position. With the advent of newer robotic systems supine surgery will be feasible in the near future.

In conclusion—the preliminary use of robotic targeting with advanced three-dimensional imaging systems enables accurate, precise, and minimally invasive stable fixation of unstable sacral fracture using minimal radiation exposure to the surgical team. Further development of these systems coupled with more extensive investigation is necessary.
